# The PrfA regulon of Listeria monocytogenes is induced by growth in low-oxygen microaerophilic conditions

**DOI:** 10.1099/mic.0.001516

**Published:** 2024-11-19

**Authors:** Lamis A. Alnakhli, Marie Goldrick, Elizabeth Lord, Ian S. Roberts

**Affiliations:** 1 School of Biology, Faculty of Biology, Medicine and Health, University of Manchester, Manchester Academic Health Science Centre, Manchester, UK

**Keywords:** *Listeria monocytogenes*, microaerophilic, PrfA regulon, RNAseq

## Abstract

*Listeria monocytogenes* is a food-borne pathogen that must adapt to several environments both inside and outside the host. One such environment is the microaerophilic conditions encountered in the host intestine proximal to the mucosal surface. The aim of this study was to investigate the expression of the PrfA regulon in response to microaerophilic growth conditions in the presence of either glucose or glycerol as a carbon source using four transcriptional (P*hly*, P*actA*, P*/prfA* and P*/plcA*) gene fusions. Further, RNAseq analysis was used to identify global changes in gene expression during growth in microaerophilic conditions. Following microaerophilic growth, there was a PrfA-dependent increase in transcription from the P*hly*, P*actA* and P*/plcA* promoters, indicating that microaerophilic growth induces the PrfA regulon regardless of the carbon source with increased expression of the PrfA, LLO and ActA proteins. A *sigB* mutation had no effect on the induction of the PrfA regulon under microaerophilic conditions when glucose was used as a carbon source. In contrast, when glycerol was the carbon source, a *sigB* mutation increased expression from the P*hly* and P*actA* promoters regardless of the level of oxygen. The RNAseq analysis showed that 273 genes were specifically regulated by microaerophilic conditions either up or down including the PrfA regulon virulence factors. Overall, these data indicated that *L. monocytogenes* PrfA regulon is highly responsive to the low-oxygen conditions likely to be encountered in the small intestine and that SigB has an input into the regulation of the PrfA regulon when glycerol is the sole carbon source.

## Introduction

*Listeria monocytogenes* is a Gram-positive facultative intracellular food-borne pathogen responsible for a number of life-threatening infections in men [1]. It has been extensively studied, and much is known about the regulation of virulence gene expression and its interaction with the host [2,4]. The virulence of *L. monocytogenes* is controlled via PrfA, a transcriptional activator from the cAMP receptor protein family [5]. The PrfA regulon consists of genes required for phagosome escape (*hly*, *plcA* and *plcB*), intracellular motility and survival in the host intestine (*actA*) and adaptation to cytosolic growth (*hpt*), together with other genes including internalins for host cell invasion [2]. The regulation of PrfA activity and expression is controlled through a number of feedback mechanisms including both transcriptional and post-transcriptional regulation as well as in response to environmental conditions [6].

During its transit through the host digestive tract, *L. monocytogenes* will be exposed to a number of environmental challenges including increased temperature, low pH and exposure to bile salts to which it will need to adapt [3]. In the intestine, there is an oxygen gradient where, at the apical mucosa near the lumen, the oxygen concentrations range from 0.1 to 1%, whereas near the vascularized submucosa, the oxygen concentration is around 6% [7]. As such, upon entering the intestine, *L. monocytogenes* will be exposed to these changes in oxygen availability and must adapt accordingly.

*L. monocytogenes* is capable of fermentative growth under anaerobic conditions, generating predominately lactate with small amounts of acetate, formate, ethanol and carbon dioxide (Romich *et al.,* 1996),[8] and it is known that it has two terminal oxidases that function at different oxygen levels [9]. Further, it has been shown that growing *L. monocytogenes* anaerobically prior to *in vitro* infection of tissue culture cells (Bo Anderson *et al*., 2007 or *in vivo* infection of gerbils increases infectivity [10][11]. In contrast, little is known about the response of *L. monocytogenes* to low-oxygen (microaerophilic) conditions that may be encountered proximal to the host mucosal surface and the role of PrfA in this process. It is known that there is an interaction between PrfA and sigmaB (SigB) and that a number of genes related to pathogenesis are coregulated by PrfA and SigB [6,12, 13]. Currently, it is unknown what role, if any, SigB might play in the adaptation to microaerophilic conditions.

In this paper, we study the expression of the PrfA regulon in response to microaerophilic growth conditions using transcriptional gene fusions and investigate the role of SigB in this process. In addition, we use RNAseq to look at global changes in gene expression during growth in microaerophilic conditions.

## Methods

### Bacterial strains and culture conditions

The bacterial strains used in this study are shown in Table 1. *L. monocytogenes* strains were grown in either tryptone soya broth (TSB; Oxoid) when constructing strains or for the physiological experiments in a defined medium (MD10) with either 49.5 mM glucose or glycerol as a carbon source as described earlier [14]. *Escherichia coli* strain DH5α was grown in Luria-Bertani (LB) broth, where necessary chloramphenicol was added to a final concentration of 7 μg ml^−1^ in TSB for the growth of *L. monocytogenes* and 35 μg ml^−1^ for the growth of DH5α. For aerobic growth, 15 ml cultures were grown in 50 ml flasks in air at 37 °C shaking at 200 r.p.m. For microaerophilic growth (oxygen 5.5%−6% v/v, carbon dioxide 10% v/v and nitrogen 85% v/v), *L. monocytogenes* were grown in a VAIN incubator (Don Whitley) in six-well plates (Costar) shaking at 140 r.p.m., respectively, at 37 °C in a culture volume of 5 ml. The OD_600_ of the culture was measured by placing 1 ml for aerobic conditions or 1 : 5 dilution for microaerophilic conditions into a cuvette and analysing the OD_600_ in a spectrophotometer (Jenway). There was no difference in the final pH of the cultures grown in MD10 media whether glucose or glycerol was used as a carbon source.

**Table 1.** Bacteria strains used in the study

Bacteria strains	Description	Reference
EGDe׃׃InlA^m^	Serotype 1/2a containing a site-directed mutation in the InlA gene to promote interaction with murine E-cadherin	[38]
*L. monocytogenes* EGDe׃׃InlA *Phly::egfp*	Chromosomal fusion of a single-copy pCG8 inserted in the InlA strain	This study
*L. monocytogenes* EGDe׃׃InlA *PactA׃׃egfp*	Chromosomal fusion of a single-copy pAD3 inserted in the InlA strain	This study
*L. monocytogenes* EGDe׃׃InlA *∆prfA*	EGDe::InlA with *prfA* deletion	This study
*L. monocytogenes* EGDe׃׃InlA *∆prfA* P*hly::egfp*	Chromosomal fusion of a single-copy pCG8 inserted in the InlA *∆prfA* mutant strain	This study
*L. monocytogenes* EGDe׃׃InlA *∆prfA PactA::egfp*	Chromosomal fusion of a single-copy pAD3 inserted in the InlA *∆prfA* mutant strain	This study
*L. monocytogenes* EGDe׃׃InlA P*/prfA::egfp*	Chromosomal fusion of a single-copy pLL1 inserted in the InlA strain	This study
*L. monocytogenes* EGDe׃׃InlA P*/plcA::egfp*	Chromosomal fusion of a single-copy pLL2 inserted in the InlA strain	This study
*L. monocytogenes EGDe׃׃InlA ∆sigB* P*hly::egfp*	Chromosomal fusion of a single-copy pCG8 inserted in the InlA *∆sigB* mutant strain	This study
*L. monocytogenes EGDe׃׃InlA ∆sigB* P*actA::egfp*	Chromosomal fusion of a single-copy pAD3 inserted in the InlA *∆sigB* mutant strain	This study
*E. coli* DH5α	*recA1*, *endA1* and *lacZΔM15*	[39]

Both the *L. monocytogenes* Δ*prfA* and Δ*sigB* mutants were constructed using the temperature-sensitive shuttle plasmid pAUL-A as described previously [4]. The plasmids pCG8, pAD3 [15,16], pLL1 and pLL2 expressing green fluorescent protein (Gfp) under the control of P*hly*, P*actA*, *prfA*P1/2 and *prfA*P3, respectively, were introduced into *L. monocytogenes* by electroporation as described previously [14]. PCR was used to confirm the chromosomal integration of the plasmids at the tRNA^Arg^-*attBB* site as described [17].

### Gfp expression

Following the growth for 25 h of the *L. monocytogenes* Gfp-reporter strains, the cells were pelleted by centrifugation at 12 000 ***g*** for 1 min and suspended in PBS to an OD_600_ of 1.0. Subsequently, 200 µl was transferred in triplicate into a black-walled 96-well plate (FALCON), and both the relative fluorescence units (RFUs; excitation at 485/20 nm and emission at 528/20 nm) and the OD_600_ were measured using the Bio-Tek Synergy HT plate reader. The results were expressed by dividing the RFU by the relative OD_600_.

### Generation of *L. monocytogenes* protein extracts

*L. monocytogenes* were grown under aerobic or microaerophilic conditions in the defined medium shaking at 37 °C for 25 h until the cultures were in the stationary phase [18]. Ten millilitres of the cultures were then centrifuged at 30 000 ***g*** and suspended in a final volume of 100 µl of buffer [167 mM Tris pH 6.8, 5.5% w/v SDS and 28% v/v glycerol]. The suspension was then boiled at 100 °C for 5 min. The samples were stored at −20 °C.

### SDS-PAGE and Western blot

To resolve listerial proteins, Tris-Glycine SDS-PAGE was performed [19]. Following the SDS-PAGE, the proteins were transferred to the PVDF membrane (Novex) using Trans-Blot Semi-Dry Transfer Cell (Bio-Rad). The transfer was performed in Tris-Glycine transfer buffer (192 mM glycine, 25 mM Tris and 20% v/v methanol) with a constant voltage of 15 V for 20 min. For PrfA and LLO protein detection, the PVDF membrane was blocked overnight at 4 °C in PBS with 4% (w/v) BSA and 0.1% (w/v) Tween 20 and followed by 1 h incubation with *L. monocytogenes* anti-PrfA rabbit polyclonal antibody or anti-LLO [20] polyclonal antibody diluted 1 : 5000 in PBS. For ActA and P60 proteins, the membrane was incubated for 2 h at room temperature with *L. monocytogenes* anti-ActA monoclonal antibody (Abnova) or anti-P60 monoclonal antibody (AdipoGen). After the incubation with the primary antibody was completed, the membrane was washed twice in PBS with 0.1% (w/v) Tween 20 before being incubated with either anti-rabbit or anti-mouse IgG HRP for 1 h at 37 °C. Finally, the membrane was washed twice in PBS with 0.1% (w/v) Tween 20 and once in PBS for 10 min prior to treatment with Plus Chemiluminescent substrate (Thermo Fisher) for 5 min. Protein bands were visualized in a ChemiDoc machine using Image Lab software.

### RNA extraction and preparation

RNA extraction was carried out using Invitrogen’s Purelink RNA Extraction Kit and Lysing Matrix E tubes (MP Biomedicals), following the manufacturer’s instructions. The Super RNAse Inhibitor (Invitrogen) was added to the samples at 1 U µl^−1^ to inhibit RNAse activity. Genomic DNA (gDNA) was removed using RNAse-free DNAse (Ambion).

Prior to sample freezing at −80 °C, small aliquots of each sample were taken to analyse for RNA integrity and concentration alongside testing for the presence of gDNA using a TapeStation System (Agilent). Agilent’s gel-supplied loading dye (5 µl) was added to 1 µl of the RNA sample in a 200 µl PCR tube. The mixture was briefly centrifuged, and the samples were heated for 3 min at 72 °C and then placed on ice for a subsequent duration of 3 min. Once cooled, the tubes were briefly vortexed and centrifuged again to remove condensation from the tube lids. The samples were transferred to the TapeStation System and analysed using the ‘Prokaryotic RNA’ and ‘No Ladder’ user settings. The RNA Integrity Number value data and associated gels were shared with the sequencing facility to ensure the high quality of samples prior to RNA sequencing.

### RNA sequencing and the result analysis

RNA sequencing was performed at the Genomic Technology Core Facility at the University of Manchester. Briefly, this involved ribosomal RNA being depleted from the samples with a ribosomal depletion kit (Illumina) as per kit instructions combined with custom probes (Integrated DNA Technologies) against the *L. monocytogenes* ribosomal RNA subunits. The RNA was then fragmented and denatured using the Illumina Stranded Total RNA Prep kit. The first and second strands were synthesized, and 3′ ends were adenylated, followed by anchor ligation, fragment and library clean-up as well as amplification. Quantitative reverse transcription PCR (KAPA) was used to quantify the libraries added to an equimolar pool which was then denatured and loaded onto a lane on the SP NovaSeq 6000 flowcell.

Unmapped paired-end sequences obtained from the HiSeq 4000 sequencer (Illumina) were tested by FastQC. Quality control included removing sequence adapters and trimming reads using the Trimmomatic V0.39 [21]. The reads were mapped against the reference genome of *L. monocytogenes* EGDe for further annotation. Counts per gene were calculated using feature Counts (subread_2.0.0 [22]). Normalization was carried out followed by principal component analysis (PCA) and differential expression (DE) calculations with the DESeq2_1.36.0 [23]. DE was defined as annotated genes of known function showing log2 fold change with significant padj <0.05 for the two replicates.

### Graphs and statistical analyses

Numerical data were tabulated into GraphPad Prism 9 software, and graphs were generated using this software. Statistical analysis was performed using GraphPad Prism 9 software. Multiple sample comparisons were analysed using ANOVA and t-test. Where appropriate, the data were analysed by a one-/two-way ANOVA using GraphPad Prism.

### Data availability

Generated sequencing data have been deposited in the ArrayExpress database at EMBL-EBI under accession number E-MTAB-12856 (https://www.ebi.ac.uk/biostudies/arrayexpress/studies/ E-MTAB-12856).

## Results

### Microaerophilic conditions induce the PrfA regulon independent of the carbon source

**Fig. 1.**
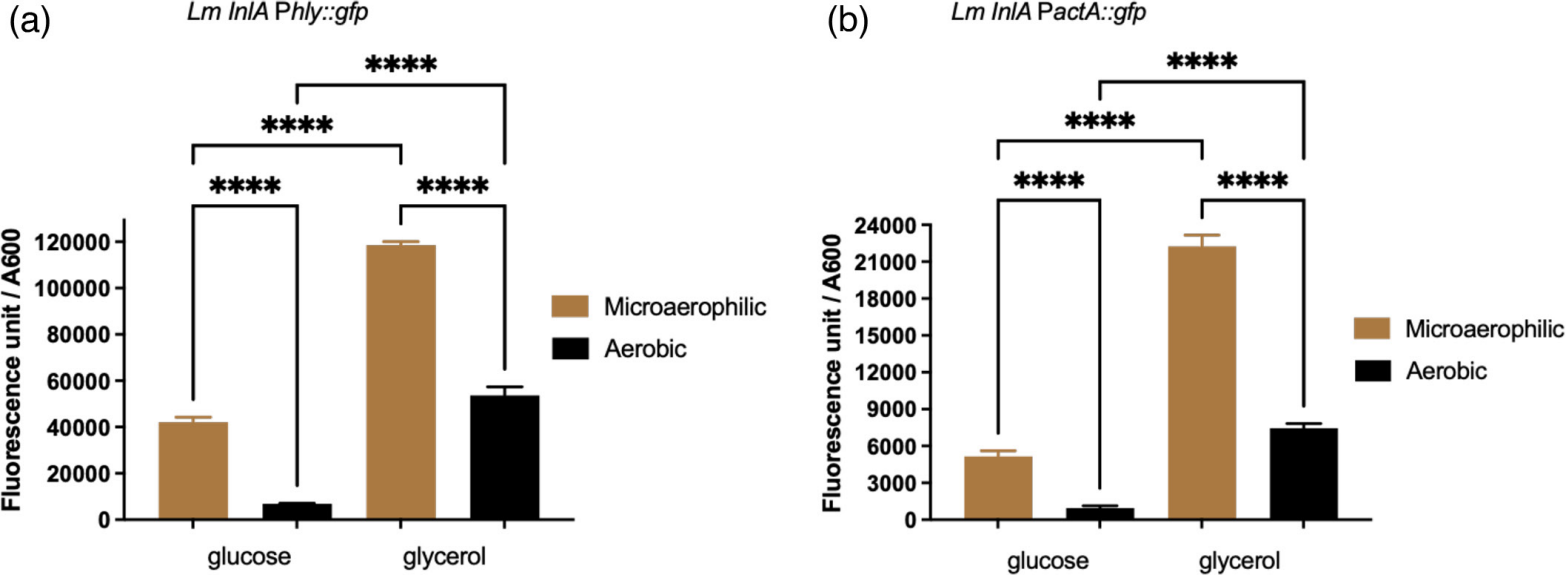
The level of Gfp expression under aerobic and microaerophilic conditions using glucose or glycerol as a carbon source in MD10 media. (a) The transcription from *phly* promoter. (b) The transcription from *pactA* promoter. The fluorescence unit divided by OD_600_ shows the transcription level of the strains. Black colour represents aerobic conditions, while brown colour represents the micraerophilic condition. The results show that microaerophilic conditions increase the Gfp expression when glucose or glycerol was used as a carbon source and able to relieve the catabolite repression of glucose. The data are the mean of three independent experiments. The error bar is the mean and sd. Significant differences (****P<0.00001) were calculated using two-way ANOVA.

To determine if transcription of the PrfA regulon that encodes many of the virulence factors of *L. monocytogenes* is induced by microaerophilic conditions, strains EGDe׃׃InlA P*hly::*e*gfp* and EGDe *inlA* P*actA::egfp* were grown aerobically and microaerophilically, and the expression of Gfp was determined. Both strains grew equally well in the MD10 media microaerophilically with either glucose or glycerol as a carbon source (*P*<0.05; Fig. S1, available in the online version of this article). In addition, both strains grew to a higher OD_600_ under microaerophilic conditions (*P*<0.05). The reasons for this increased growth under microaerophilic conditions when either carbon source is used are currently unknown. Following microaerophilic growth, there was a significant increase in transcription from both P*hly* and P*actA* promoters compared to aerobic conditions regardless of the carbon source used (Fig. 1). Expression of the P*hly* was 6962 RFU/OD_600_ following aerobic growth in glucose but increased to 42129 RFU/OD_600_ following microaerophilic growth; while in glycerol, it reached 53660 RFU/OD_600_ under aerobic conditions compared to 118589 RFU/OD_600_ under microaerophilic conditions. Expression of the P*actA* was low 955 RFU/OD_600_ following aerobic growth in glucose but increased to 5144 RFU/OD_600_ following microaerophilic growth, whereas in glycerol, it reached 7453 RFU/OD_600_ under aerobic conditions compared to 22247 RFU/OD_600_ under microaerophilic conditions. The increase in expression when glycerol was the carbon source is in keeping with the known inhibition of PrfA by the phosphorylation status of the phosphotransport uptake system (PTS) [24].

### The induction of the P*hly* and *P*actA promoters following microaerophilic growth is PrfA-dependent

**Fig. 2.**
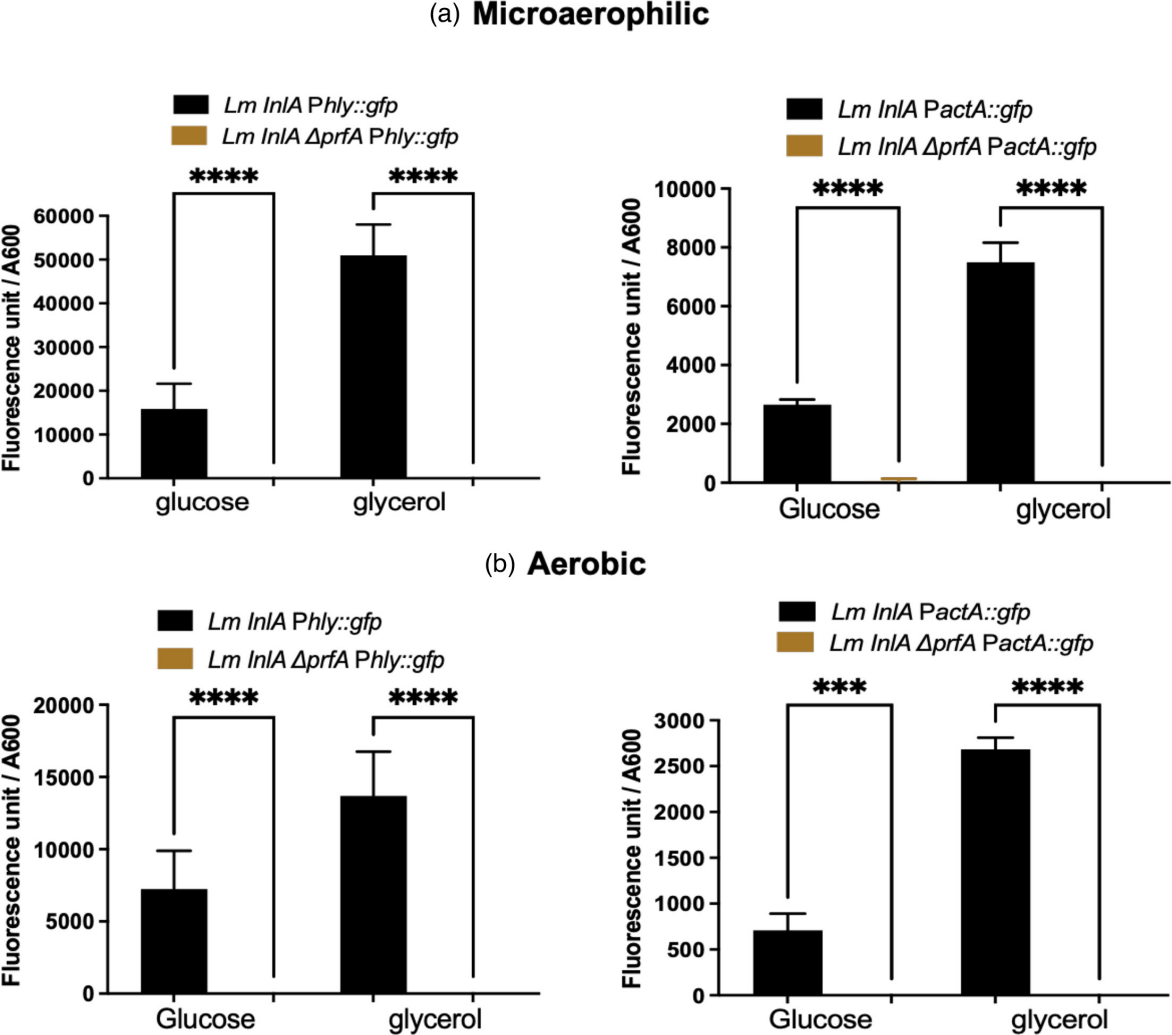
The effect of a *prfA* mutation on the level of expression in either glucose or glycerol as a carbon source in MD10 media. (a) The transcription in microaerophilic conditions. (b) The transcription in aerobic conditions. The fluorescence unit divided by OD_600_ shows the transcription level of the strains. Four strains were used in experiments: two as a control in black colour (Lm InlA *phly::Gfp* and Lm InlA *pactA::Gfp*) and the other two with *prfA* mutation in brown colour (Lm InlA *∆prfA phly::Gfp* and Lm InlA *∆prfA pactA::Gfp*). The results revealed that *prfA* mutants abolished detectable transcription from *phly* and *pactA* when glucose or glycerol was used as a carbon source under aerobic or microaerophilic conditions. The data are the mean of three independent experiments. The error bar is the mean and sd. Significant differences (***P<0.0001; ****P<0.00001) were calculated using one-way ANOVA.

**Fig. 3.**
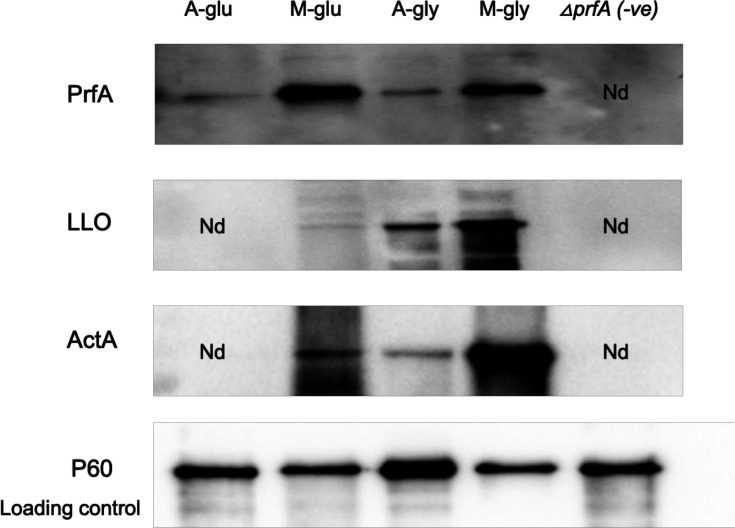
Representative expression of PrfA-regulated proteins under different growth conditions. The protein was extracted from *L. monocytogenes* EGDe׃׃InlA^m^ grown in MD10 medium and analysed by Western blotting with anti-PrfA, anti-LLO, anti-ActA and anti-P60 antibodies. A-glu and A-gly mean (under aerobic condition using glucose or glycerol as a carbon source, respectively), M-glu and M-gly mean (under microaerophilic condition using glucose or glycerol as a carbon source, respectively) and Nd (no detected band). *∆prfA* was used as a negative control, and P60 levels were used as a loading control.

To confirm that the observed increase in transcription from the P*hly* and P*actA* promoters under microaerophilic conditions was PrfA-dependent, the experiment was repeated with *prfA* mutants. The results revealed that *prfA* mutants abolished detectable transcription from P*hly* and P*actA* when glucose or glycerol was used as the carbon source under aerobic or microaerophilic conditions confirming the key role of PrfA in inducing transcription in microaerophilic conditions (Fig. 2). To confirm that the increased levels of transcription were reflected in increased LLO and ActA expression, lysates of cultures grown under aerobic and microaerophilic conditions were Western blotted using LLO- and ActA-specific antisera. As a loading control, the levels of the P60 protein [25], a protein whose expression does not alter under microaerophilic conditions [18], were detected (Fig. 3). The blot showed that under microaerophilic conditions, regardless of the carbon source, there was a significant increase in the levels of LLO and ActA proteins (Fig. 3).

### Microaerophilic growth induces increased transcription of the *prfA* gene that results in increased PrfA protein

**Fig. 4.**
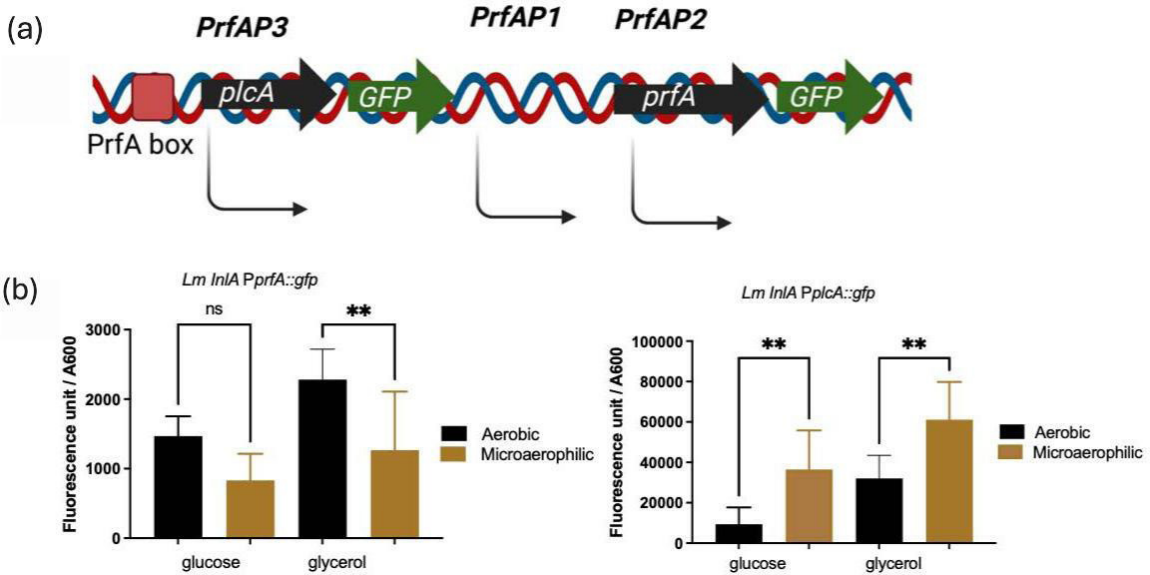
The transcription from PrfA promoters under aerobic and microaerophilic conditions using glucose or glycerol as a carbon source in MD10 media. (a) The operon structure and promoter organization of the *plcA-prfA* locus involve the expression of PrfA. (b) The transcription from *p/prfA* and *p/prfA* promoters. The fluorescence unit divided by OD_600_ shows the transcription level of the strains. The black colour shows the transcription under aerobic conditions, while brown colour shows the transcription under microaerophilic conditions. The error bar is the mean and sd. Significant differences (ns, non-significant; **P<0.05) were calculated using two-way ANOVA.

PrfA activity is regulated through a number of feedback mechanisms including both transcriptional and post-transcriptional regulation [6]. Transcriptional regulation of *prfA* is mediated by three promoters (Fig. 4a), two of which, *prfA*P1 and *prfA*P2, are immediately upstream of *prfA* and a third promoter *prfA*P3 is upstream of the *plcA* gene [6,26]. Transcription from *prfA*P1 and *prfA*P2 results in the initial synthesis of PrfA to activate transcription of the *hly* and *plcA* genes for phagosomal escape [27]. Activation of the *prfA*P3 promoter results in the expression of both the *plcA* and *prfA* genes and higher levels of PrfA [26]. To determine if the increase in expression of the PrfA regulon is a consequence at least in part due to increased transcription of *prfA*, two transcriptional fusions to *gfp* were generated (Fig. 4a). First, a PCR fragment, containing *prfA*P1 and *prfAP2*, was cloned into the integrative vector pPL2 [16] to generate strain EGDe׃׃InlA P/*prfA::egfp*. Second, the *prfA*P3 promoter was cloned into the same vector to generate strain EGDe׃׃InlA P***/****plcA::egfp*. Growth of strain EGDe׃׃InlA P*/prfA::egfp* with glucose as a carbon source resulted in no significant difference in Gfp expression between aerobic and microaerophilic conditions (Fig. 4b). When glycerol was used as a carbon source, there was a significant decrease in Gfp expression following microaerophilic growth (Fig. 4b). In contrast, following microaerophilic growth of strain EGDe׃׃InlA P***/****plcA::egfp*, there was a significant increase in Gfp expression as compared to aerobic growth regardless of the carbon source used (Fig. 4b). This indicates that increased *prfA* transcription in microaerophilic conditions is being driven by the *prfA*P3 promoter. To confirm that these changes in transcription were reflected in PrfA protein levels, Western blotting was performed on culture lysates grown under different conditions (Fig. 3). The blot shows that growth in microaerophilic conditions results in increased PrfA levels compared to those detectable following growth in aerobic conditions, indicating that the increase in *prfA* transcription following microaerophilic growth also resulted in increased PrfA expression.

### SigB affects transcription from both P*hly* and P*actA* under both aerobic and microaerobic conditions when glycerol is used as a carbon source

**Fig. 5.**
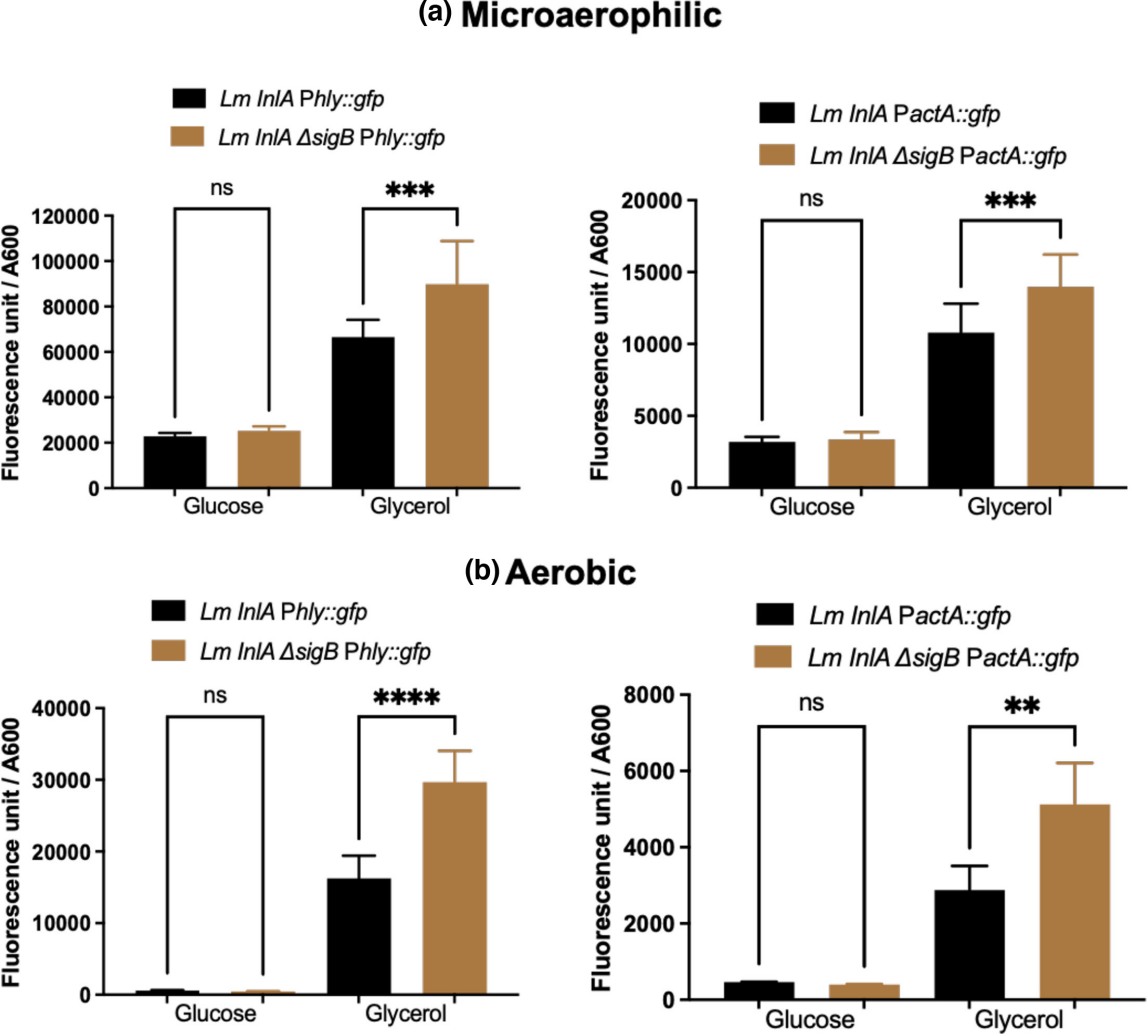
The effect of a *sigB* mutation on the level of expression in either glucose or glycerol as a carbon source in MD10 media. (a) The transcription in microaerophilic conditions. (b) The transcription in aerobic conditions. The fluorescence unit divided by OD_600_ shows the transcription level of the strains. Four strains were used in experiments: two as a control in black colour (*Lm InlA phly::Gfp* and *Lm InlA pactA::Gfp*) and the other two with *sigB* mutation in brown colour (*Lm InlA ∆sigB phly::Gfp* and *Lm InlA ∆sigB pactA::Gfp*). The results revealed that *sigB* mutants increased the transcription from *phly* and *pactA* when glycerol was used as a carbon source regardless of the oxygen conditions. The data are the mean of at least three independent experiments. The error bar is the mean and sd. Significant differences (**P<0.001; ***P<0.0001; ****P<0.00001) were calculated using one-way ANOVA.

It is known that there is an interaction between PrfA and sigmaB (SigB), that the *prfA*P2 promoter is regulated in part by SigB and that a number of genes related to pathogenesis are coregulated by PrfA and SigB [6,12, 13]. To investigate if SigB was involved in the adaptation to microaerophilic growth, strains EGDe׃׃InlA ∆*sigB* P*hly::*e*gfp* and EGDe *inlA****∆****sigB* P*actA:׃egfp* were grown in MD10 media aerobically and microaerophilically with glucose as a carbon source. Both strains grew to a similar final OD_600_ as the wild-type strains albeit with a slight lag at the start of growth (Fig. S2). Following growth in either aerobic or microaerophilic conditions with glucose as a carbon source, the *sigB* mutation had no effect on the levels of Gfp expression (Fig. 5). In contrast, when glycerol was used as a carbon source, the *sigB* mutation resulted in a slight decrease in growth rate as reported previously for *sigB* mutant utilizing glycerol as a sole carbon source [28] but still reaching the similar final OD_600_ as the wild-type strains (Fig. S2). The *sigB* mutation increased Gfp expression in strains EGDe׃׃InlA ***∆****sigB* P*hly::*e*gfp* and EGDe *inlA****∆****sigB* P*actA׃׃egfp* in both aerobic and microaerophilic growth conditions (Fig. 5). These data indicate that SigB or a SigB-regulated gene is either directly or indirectly repressing both the P*hly* and P*actA* promoters when glycerol is used as a carbon source regardless of the oxygen levels.

Taken as a whole, these data show that following growth microaerophilically, there is a PrfA-dependent increase in transcription from both the P*hly* and P*actA* promoters indicating that microaerophilic growth induces the PrfA regulon. This induction occurs even in the presence of glucose that inhibits PrfA activity.

### RNAseq analysis of microaerophilic-grown *L. monocytogenes* with glucose or glycerol as a carbon source

**Fig. 6.**
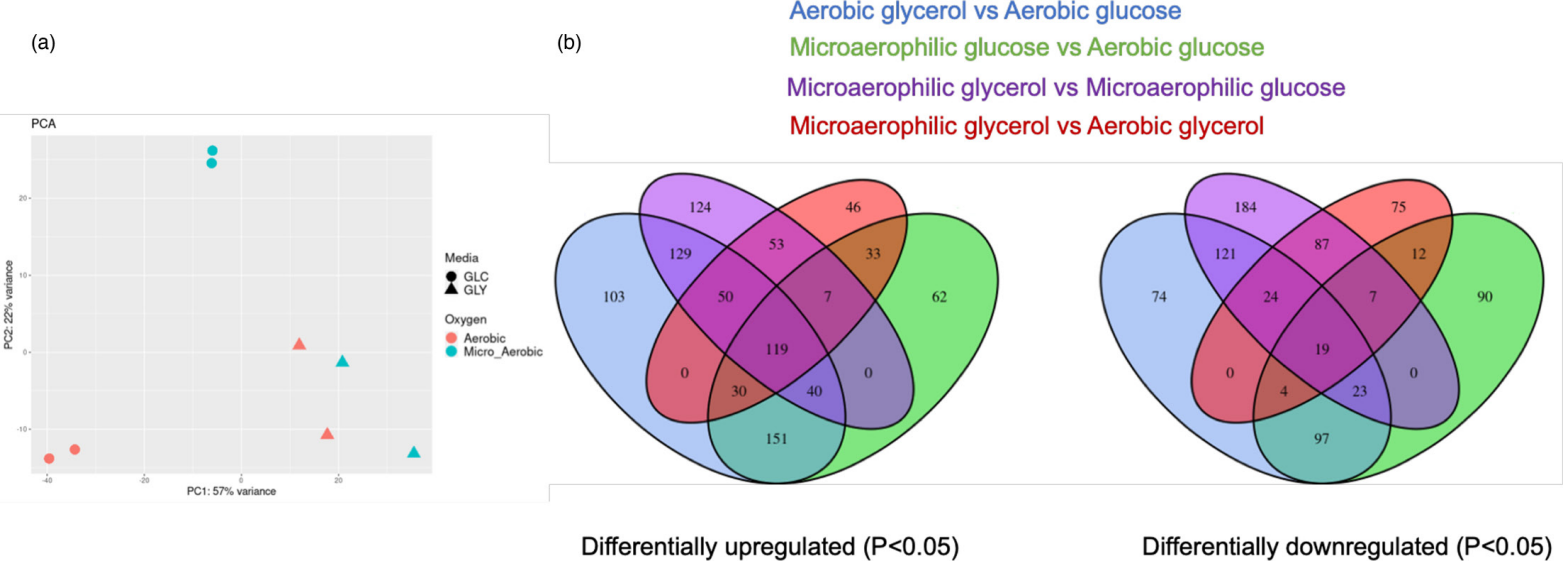
Transcriptomic analysis of *L. monocytogenes* under microaerophilic or aerobic conditions using glucose or glycerol as a carbon source in MD10 media. (a) PCA of gene expression for *L. monocytogenes inlA* in microaerophilic and aerobic conditions using glycerol or glucose as a carbon source. All four conditions were replicated twice. Each data point represents one replicate. Aerobic conditions are represented in red, and microaerophilic conditions are represented in green. When glycerol was used as a carbon source, this is represented by triangles, and when glucose was used as a carbon source, this is shown by circles. Variance explained by PC1 and PC2 is shown on the *x*-axis and *y*-axis, respectively. (b) The Venn diagram showing overlapping gene sets between the analysed conditions. The Venn diagram on the left shows genes differentially upregulated, while the Venn diagram on the right shows genes differentially downregulated. The blue group represents genes differentially expressed in aerobic conditions using glycerol as a carbon source compared to aerobic conditions using glucose as a carbon source. Green represents genes differentially expressed in microaerophilic conditions using glucose as a carbon source compared to aerobic conditions using glucose as a carbon source. Purple represents genes differentially expressed in microaerophilic conditions using glycerol as a carbon source compared to microaerophilic conditions using glucose as a carbon source. Red represents genes differentially expressed in microaerophilic conditions using glycerol as a carbon source compared to aerobic conditions using glycerol as a carbon source. The numbers indicate the number of genes, and the overlaps highlight the number of gene sets that are shared between conditions.

To investigate the changes in the global gene expression, RNAseq was performed on *L. monocytogenes* InlA following growth under microaerophilic and aerobic conditions using either glucose or glycerol as a carbon source. In the PCA for media and oxygen, there was robust separation among the different conditions (Fig. 6a). Specifically, the first principal component (PC1) representing the oxygen concentration accounted for 57% of the variance, and the second principal component (PC2) representing carbon source accounted for 22% of the variance. All conditions showed robust separation, and oxygen type (microaerophilic or aerobic) caused a larger effect on gene expression than media glucose and glycerol.

A Venn diagram of overlapping gene sets assigned to pairwise comparisons is presented in Fig. 6(b), in which the *P*-value for all genes was <0.05. Under microaerophilic conditions with glucose as a carbon source, there were 62 genes specifically upregulated, and 90 were downregulated (Fig. 6b, shown in green). In contrast, there were 46 genes specifically upregulated, and 75 were downregulated under microaerophilic conditions with glycerol as a carbon source (Fig. 6b, shown in red). These genes are those exclusively regulated by microaerophilic conditions. In addition, there were 232 upregulated genes that were shared with glucose or glycerol as a carbon source, while 349 were downregulated in response to microaerophilic conditions (Fig. 6b, shown in the green-purple-red overlap). Interestingly, this set of shared genes also includes genes from the PrfA regulon which were upregulated in microaerophilic conditions regardless of the carbon source that was used. It should be noted that regardless of the growth conditions, the change of carbon source induced significant transcriptional changes. For instance, 103 genes were significantly upregulated under aerobic conditions when glycerol was used as a carbon source compared to glucose (Fig. 6b, shown in blue). However, what is clear is that microaerophilic conditions induce significant transcriptional change in *L. monocytogenes* compared to aerobic growth.

### Differentially expressed genes in microaerophilic conditions specific to glucose as a carbon source

**Fig. 7.**
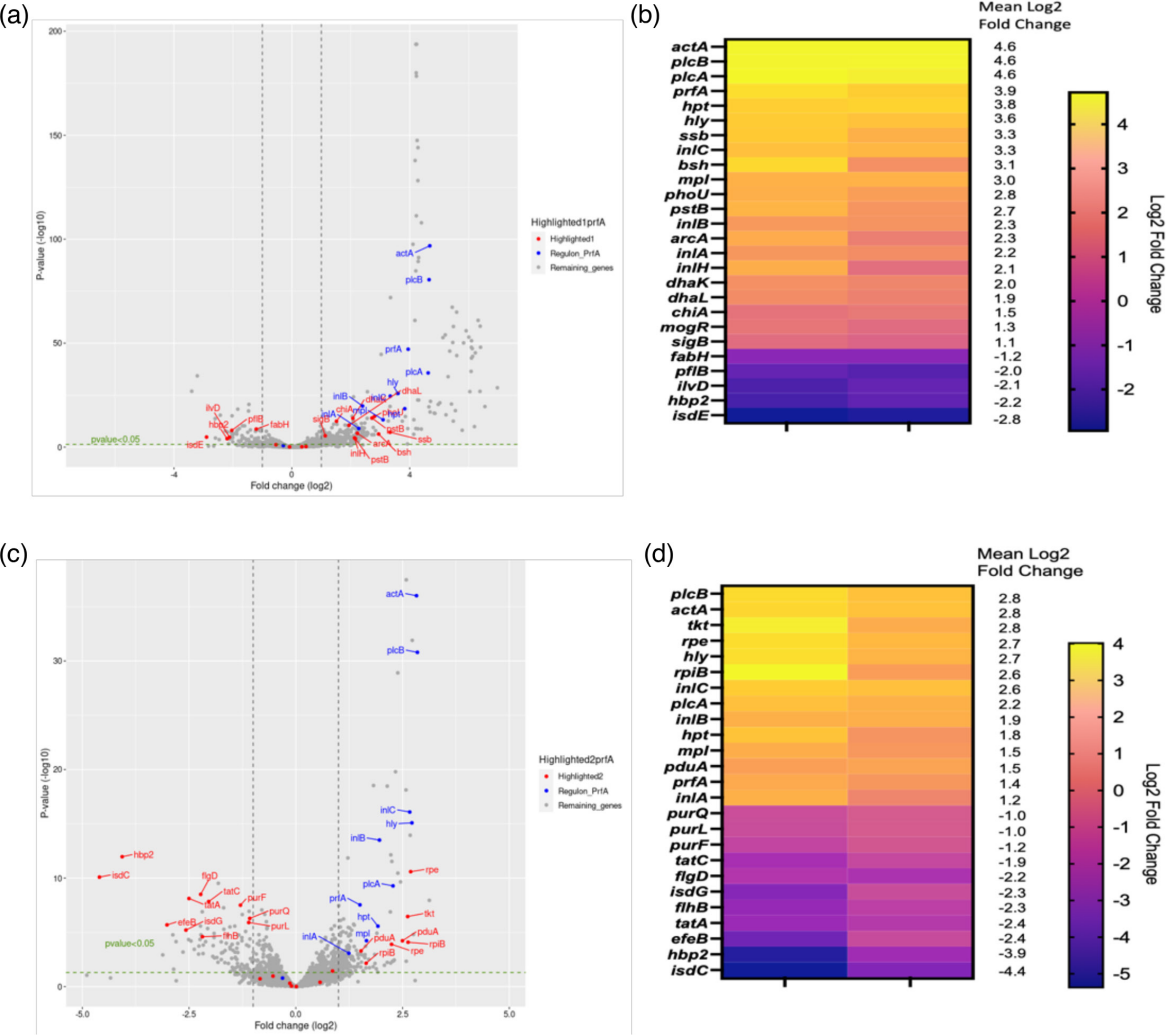
RNAseq analysis of *L. monocytogenes* under microaerophilic versus aerobic conditions using glucose or glycerol as a carbon source in MD10 media. (a, b) Genes that have significant transcriptomic changes under microaerophilic conditions specific to glucose as a carbon source. (c, d) Genes with significant transcriptomic changes under microaerophilic conditions specific to glycerol as a carbon source. (a, c) The volcano plot showing the differential gene expression in two biological replicates under microaerophilic versus aerobic conditions. An individual gene is represented by circles, while filtering differentially expressed genes is represented by dotted lines. Circles in red show annotated genes of known function that are significantly down/upregulated, while circles in blue show PrfA regulon genes that are all significantly upregulated. (b, d) A heat map showing log2 fold change of annotated genes of known function with significant padj <0.05 for two replicates. Gene values are ordered from highest to lowest fold change, from top to bottom. The key indicates the log2 fold change values for each colour.

RNAseq analysis identified a total of 694 significantly differentially expressed genes above log2 fold change in microaerophilic conditions compared to aerobic conditions when glucose was used as a carbon source (Fig. 7a). A total of 442 genes were downregulated, and 252 genes were upregulated, of which 151 had known functions. The log2 fold changes and names of the most highly up/downregulated characterized genes are shown on a heat map (Fig. 7b).

The heat map shows log2 fold change values for 26 characterized genes that had a mean of log2 fold change >1 or <-1. Most of these genes were upregulated; for example, *ssb*, which encodes for single-stranded DNA-binding protein, had a mean log2 fold change of 3.36; *bsh*, which encodes for choloylglycine hydrolase, had a mean log2 fold change of 3.31; *phoU*, which encodes for phosphate signalling complex protein PhoU, had a mean log2 fold change of 2.81. However, five of these genes were downregulated, which include *isdE*, *hbp2*, *ilvD*, *pflB* and *fabH* that had −2.89, −2.20, −2.1, −2.0 and −1.20 mean log2 fold changes, respectively.

The most upregulated genes under microaerophilic conditions were the PrfA-regulated virulence genes (Fig. 7b). All of the PrfA regulon genes were upregulated as expected (Fig. 7a, highlighted in blue). The log2 fold change of *actA* and *plcB/A* was the highest, with a mean log2 fold change of 4.6 across the replicates, and the mean log2 fold change of *inlB* was 2.3 and 2.2 for *inlA,* which both showed the lowest values.

### Differentially expressed genes in microaerophilic conditions specific to glycerol as a carbon source

RNAseq analysis identified a total of 566 significantly differentially expressed genes above log2 fold change in microaerophilic conditions compared to aerobic conditions when glycerol was used as a carbon source (Fig. 6c). A total of 338 genes were downregulated, and 228 genes were upregulated, of which 89 had known functions. The log2 fold changes and names of the most highly up/downregulated characterized genes are shown on a heat map (Fig. 7d).

The heat map shows log2 fold change values for 15 characterized genes that had a mean of log2 fold change >1 or <-1. Most of these genes were downregulated; for example, *isdC*, *isdG*, which encodes for haem oxygenase, and *isdC*, was the highest mean log2 fold change of −4.4, while *isdG* was −2.35. *hbp2*, which encodes for haemoglobin-binding protein Hbp2, was the second highest mean log2 fold change of −3.92. *tatC* and *tatA* are genes in twin-arginine translocation pathway and had log2 fold change of −1.97 and −2.35, respectively. *purF*, *purL* and *purQ*, which encode for phosphoribosyl formyl glycin amidine synthase group, showed log2 fold change of −1.2, −1.0 and −1.0, respectively. In contrast, the PrfA-regulated virulence genes were the most genes upregulated (Fig. 7c, highlighted in blue) when glycerol was used as a carbon source. Similar to the PrfA regulon when glucose was used as a carbon source, all of the PrfA regulon genes were upregulated. The log2 fold change of *actA* and *plcB* was the highest, with a mean log2 fold change of 2.8 across the replicates, and the mean log2 fold change of *inlA* was 1.2 which showed the lowest value. Overall, all the values showed a statistically significant (padj <0.05) log2 fold increase. Using the log2 fold change >1 or <-1 criterion, there were four additional genes that were upregulated including *pduA* with a log2 fold change of 1.53, and *rpiB*, *rpe* and *tkt* which all had an average of 2.7 log2 fold change.

## Discussion

In this paper, we identify for the first time that microaerophilic conditions, likely to be encountered proximal to the epithelial surface in the intestine, induce the PrfA regulon. Transcription of both the *hly* and the *actA* genes was induced following growth in microaerophilic conditions which resulted in increased LLO and ActA expression. This demonstrates that genes that are switched on initially by lower levels of PrfA such as *hly* and those that are induced subsequently by higher levels of PrfA such as *actA* [26] are induced in microaerophilic environments. Transcriptional fusions to either the *prfA*P3 or *prfA*P1/2 promoters that regulate transcription of *prfA* demonstrated that under microaerophilic conditions, there was increased transcription of the *prfA* gene that was driven predominantly from the *prfA*P3 promoter with barely detectable transcription from the P*/prfA* promoter region (Fig. 4a). The *prfA*P3 promoter upstream of *plcA* is activated by PrfA and induces expression of the bicistronic *plcA-prfA* transcript leading to higher levels of PrfA [29], while it is unclear how other post-transcriptional regulatory pathways that are known to regulate the availability of PrfA are affected under microaerophilic conditions. It is clear that the increased transcription of the *prfA* gene under these conditions results in increased PrfA which in turn activates the PrfA regulon. At this stage, it is unknown how the transcription of the *prfA* gene is regulated in response to microaerophilic conditions. Under strict anaerobic conditions, it has been shown that while there is increased *hly* transcription, there is not a concomitant increase in LLO suggesting a role for oxygen in LLO production [30]. Clearly, under the microaerophilic conditions used in our study, this block in production for LLO and ActA proteins was not seen, suggesting that the low levels of oxygen present in microaerophilic conditions are sufficient. Since it has been shown that ActA expression in the gut promotes biofilm formation and bacterial persistence in the host [31], the induction of the PrfA regulon by microaerophilic conditions could promote increased persistence of *L. monocytogenes* in the host. It should also be appreciated that such microaerophilic conditions could be encountered in silage if incomplete fermentation has occurred [32]. As such, contamination of silage with *L. monocytogenes* under such conditions could induce the PrfA regulon, potentially increasing the risk of listeriosis to animals that may subsequently consume the silage.

The induction of the PrfA regulon by microaerophilic conditions occurred regardless of whether glucose or glycerol was used as a carbon source albeit at higher levels in the presence of glycerol (Figs 1 and 7). Although the levels of PrfA appeared higher in microaerophilic grown cultures when glucose rather than glycerol was the carbon source, there was a more modest increase in transcription of the PrfA regulon (Figs 1 and 8). We believe that this is a consequence of the known inhibition of PrfA activity by the phosphorylation status of the PTS uptake system for the import of glucose [24]. However, the increased transcription of the PrfA regulon in the presence of glucose would indicate that in microaerophilic conditions, the increased expression of PrfA is at least in part able to overcome this inhibition. The RNAseq data showed that expression of the *gshF* gene was unaltered during microaerophilic growth. GshF is the glutathione synthase responsible for the production of intracellular glutathione for post-translational activation of PrfA [33]. As such, this would indicate that increased PrfA activity is not driven by increased glutathione production during microaerophilic growth. The observation that a *sigB* mutation resulted in increased transcription of the *hly* and *actA* genes when glycerol was used as a carbon source regardless of the oxygen levels was surprising. SigB is an alternative sigma factor that drives the expression of genes essential for stress survival [34], and there is an interplay between the SigB and PrfA regulons [29]. These results would indicate a role for SigB, or a SigB-regulated gene, in the repression of the PrfA regulon when glycerol is used as a carbon source. Although it has been shown that SigB activity is influenced by the carbon source [35], to our knowledge, this is the first report of a linkage between the carbon source, SigB and the PrfA regulon. Although the levels of glycerol used in these studies are in keeping with those used in previous studies [28,35, 36], it should be appreciated that glycerol is a three-carbon sugar as compared to glucose that is a six-carbon sugar. As such, in terms of available carbon, there is half the amount in those cultures supplemented with glycerol. However, this does not detract from the observations regarding the induction of the PrfA regulon by microaerophilic conditions.

Of the 134 genes that showed a log2 fold change in expression either up or down when comparing glycerol- versus glucose-grown cells in aerobic conditions, 26 had previously been identified when glycerol was used as a carbon source [36]. Among these 26 genes were those that were upregulated when glycerol was the carbon source such as the PrfA regulon, genes for glycerol metabolism such as *glpK* and *dhak* [37] and those strongly downregulated such as the purine biosynthetic pathway (supplementary data). The differences between the RNAseq data here and that of the previous micro-array data probably reflect the RNAseq gives a much better quantitative range of gene expression and identifies greater differentially regulated genes.

In summary, the data in this paper establish that the PrfA regulon is responsive to low-oxygen conditions, which results not only in increased transcription of the regulon but also in protein expression. The significance of this induction of the PrfA regulon in conditions likely encountered proximal to the gut epithelium is that it will prime the bacterium ahead of any possible interactions with the host mucosa with increased expression of ActA-promoting persistence in the host gut. In future studies, it will be exciting to probe the mechanism of this regulation of the PrfA regulon by microaerophilic conditions. The observation that, regardless of the oxygen levels, when glycerol is the sole carbon source, SigB appeared to be directly or indirectly repressing the P*hly* and P*actA* promoters is a new role that warrants further investigation.

## supplementary material

10.1099/mic.0.001516 Uncited Fig. S1.

## Abbreviations

DE: differential expression
gDNA: genomic DNA
Gfp: green fluorescent protein
PCA: principal component analysis
RFUs: relative fluorescence units
TSB: tryptone soya broth
