# Supplementary material for: The PrfA regulon of Listeria monocytogenes is induced by growth in low-oxygen microaerophilic conditions
Source: Microbiology (Reading). 2024 Nov 19;170(11):001516. doi: 10.1099/mic.0.001516 (PMC11575702; doi:10.1099/mic.0.001516)
Supplement: Uncited Fig. S1. [file mic-170-01516-s001.pdf]

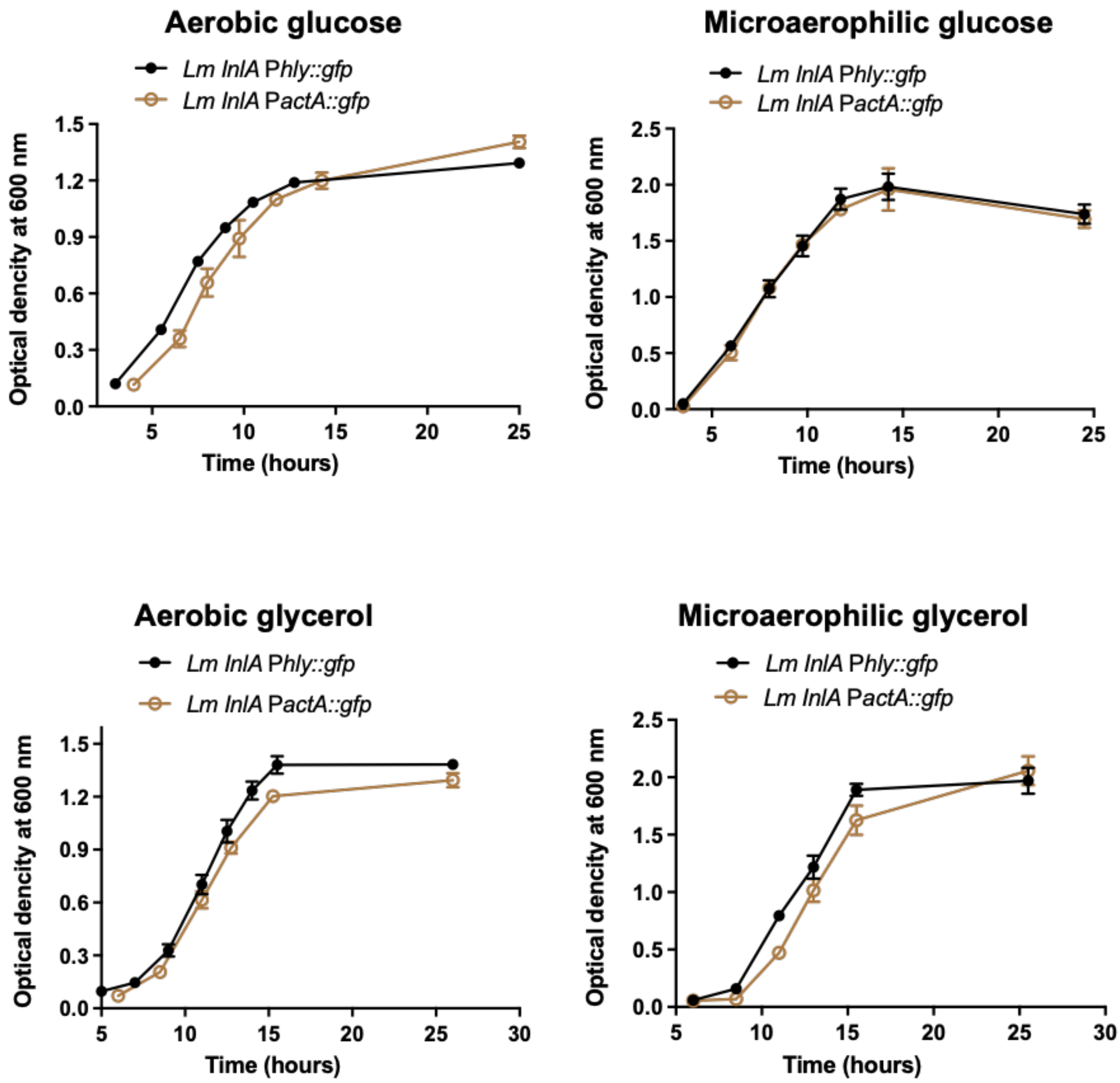

Figure S1 The growth curves of *L. monocytogenes* InlA strains grown aerobically or microaerobically using glucose and glycerol as a carbon source. Means of triplicates were plotted with error bars representing standard deviation. The result is the mean of three independent experiments.

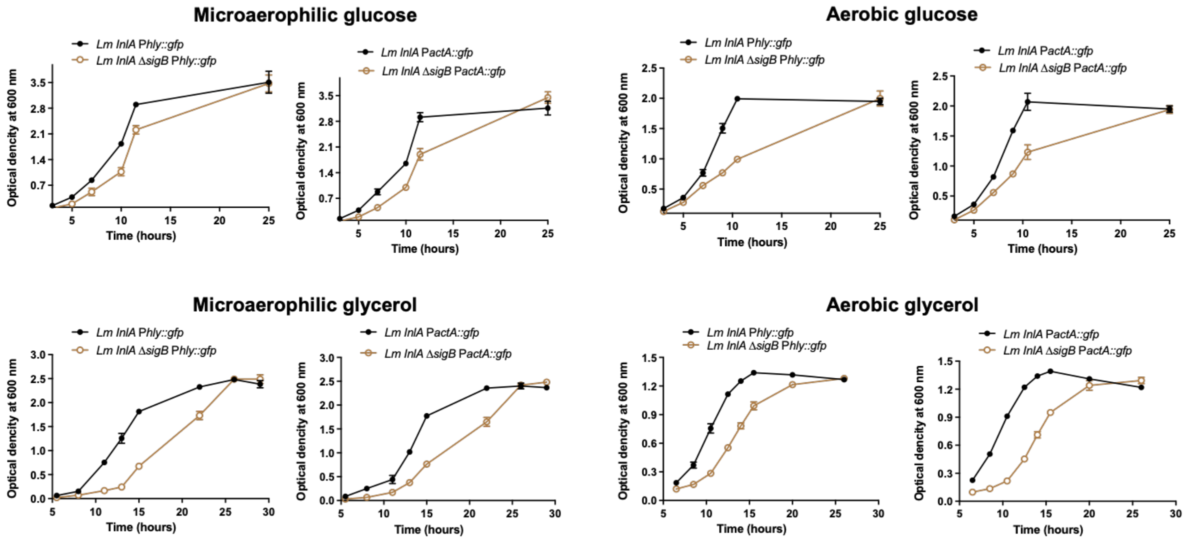

Figure S2 The effect of a *sigB* mutation on the growth of *L. monocytogenes* using glucose or glycerol as carbon source under aerobic and microaerobic conditions. Means of triplicates were plotted with error bars representing standard deviation. The result is the mean of three independent experiments.

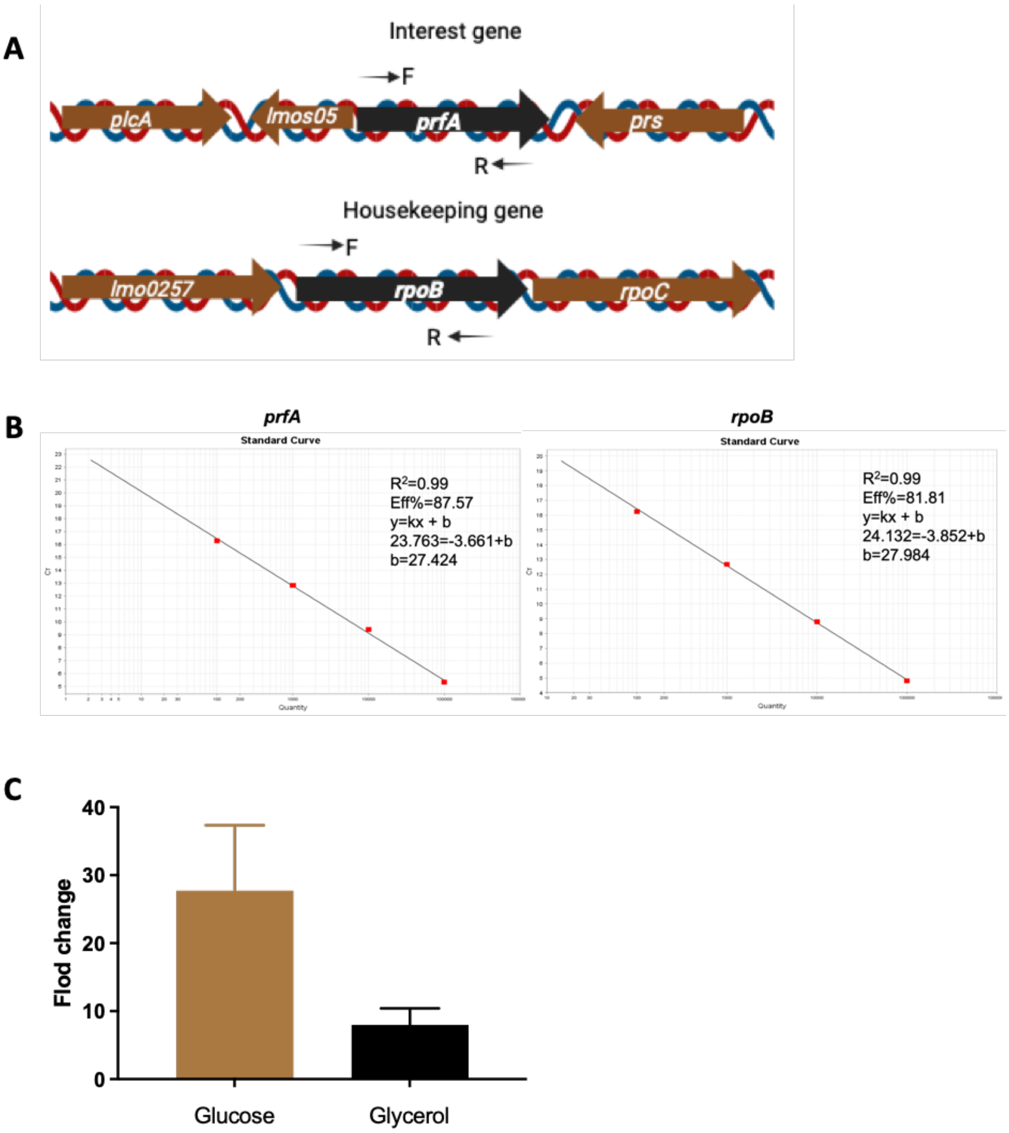

Figure S3 A Illustration of the primers used in qrtPCR and their corresponding amplification. The figure shows the *prfA* and *rpoB* genes and the location of the primers used to amplify products for the qrtPCR. **B shows standard curves for *prfA* and *rpoB* amplicons performed in qrtPCR assay.** The graphs were generated by plotting the CT values against the logarithm of the initial copy numbers using StepOneTM software v2.3. X-axis unit is copies/µl. The R2 is the regression coefficient calculated from the regression line. The R^2 value indicates the closeness of fit between the standard curve regression line and the individual CT data points from the standard reactions. A value of 1 indicates that the regression line is perfectly fitted to the data points. The Eff% represents the efficiency of amplification. The linear regression equation is shown on each standard curve as y=kx+b. An y-intercept indicates the predicted CT value for a sample (x) with a quantity equal to 1, and K is the slope of the curve. From the given values, the b was calculated for each amplicon using the linear regression equation. **C shows fold changes in expression of *prfA* under microaerobic conditions against aerobic conditions using either glucose or glycerol as a carbon source.** RNA was extracted at OD_{600} =0.5 for *L. monocytogenes InlA* strain grown in MD10 using glucose or glycerol as a carbon source. qrtPCR was performed using the primer sets mentioned in Figure 5.1. Values are the mean of three independent experiments normalised against *rpoB* transcripts and aerobic conditions. Error bars represent the standard error of the mean. (p value 0.025) using t test.
